# Increased Mortality Risk in People with Type 2 Diabetes Mellitus in Lithuania

**DOI:** 10.3390/ijerph17186870

**Published:** 2020-09-20

**Authors:** Donata Linkeviciute-Ulinskiene, Auguste Kaceniene, Audrius Dulskas, Ausvydas Patasius, Lina Zabuliene, Giedre Smailyte

**Affiliations:** 1 Institute of Biomedical Sciences, Department of Pathology, Forensic Medicine and Pharmacology, Faculty of Medicine, Vilnius University, Ciurlionio g. 21, 03101 Vilnius, Lithuania; 2 Laboratory of Cancer Epidemiology, National Cancer Institute, P. Baublio g. 3b, 08406 Vilnius, Lithuania; auguste.kaceniene@gmail.com (A.K.); audrius.dulskas@nvi.lt (A.D.); ausvydas.patasius@nvi.lt (A.P.); giedre.smailyte@nvi.lt (G.S.); 3 Department of Abdominal and General Surgery and Oncology, National Cancer Institute, Santariskiu g. 1, 08406 Vilnius, Lithuania; 4 Institute of Health Sciences, Faculty of Medicine, Vilnius University, Ciurlionio g. 21, 03101 Vilnius, Lithuania; 5 Institute of Clinical Medicine, Faculty of Medicine, Vilnius University, Santariskiu g. 2, 08406 Vilnius, Lithuania; lina.zabuliene@mf.vu.lt

**Keywords:** type 2 diabetes mellitus, mortality, standardized mortality ratio, population study, retrospective cohort study.

## Abstract

This retrospective cohort study aimed to analyze overall and cause-specific mortality risk in people with type 2 diabetes mellitus (T2DM) in Lithuania. Information on the diagnosis of T2DM and glucose-lowering medication was obtained from the National Health Insurance Fund database, causes of death–from death certificates. Sex, age, and calendar period-standardized mortality ratios (SMRs) were calculated. In addition, 89,512 patients were followed-up between 2010 and 2017, contributing to the observation period of 592,321 person-years. Overall mortality risk was increased for both sexes (overall SMR = 1.35, 95% confidence interval (CI) 1.34–1.37). Greatest mortality risk was in the age group of 40–49 years at diabetes diagnosis (SMR = 1.68, 95% CI 1.60–1.76) and among those who had died before the age of 50 (SMR = 22.04, 95% CI 18.82–25.81). Patients treated with insulin only had the highest SMR (2.43, 95% CI 2.32–2.55). Mortality risk increased with increasing diabetes duration and was higher in women in all these groups. The highest cause-specific SMRs were infection-related causes (SMR = 1.44), particularly septicemia (SMR = 1.78), diseases of the circulatory system (SMR = 1.42), especially ischemic heart (SMR = 1.46) and cerebrovascular diseases (SMR = 1.38), as well as diseases of the digestive system (SMR = 1.35). Cancer mortality risk was elevated for women (SMR = 1.13), but not for men (SMR = 0.93). In conclusion, people with T2DM had an excess mortality risk, which was higher in women compared to men, younger people, in those who were diagnosed with T2DM at a younger age, had longer diabetes duration, and who required treatment with insulin.

## 1. Introduction

Type 2 diabetes mellitus (T2DM) and its complications have been an increasing burden of mortality and disability globally. The Global Burden of Disease Study 2013 identified diabetes as the ninth major cause of reduced life expectancy [1]. Furthermore, it was estimated that diabetes caused 4.2 million deaths in adults aged 20–79 years during 2019, which is 11.3% of global mortality. In addition, nearly half of these deaths (46.2%, 1.9 million) are estimated to occur in working-age adults, younger than 60 years [2].

The increased risk for (and death from) cardiovascular diseases (CVD) in patients with T2DM worldwide is well-established [3,4,5]. Diabetes is also among the leading causes of kidney failure [6,7]. Excess mortality due to infection-related diseases in patients with diabetes has been demonstrated as well [8]. Furthermore, diabetes has been associated with an increased risk for some types of site- specific cancer [9]. In fact, we recently found that T2DM is associated with increased risk for pancreas, liver, kidney, and thyroid cancer in men and women and breast and corpus uteri in women in the Lithuanian population [10]. Whether cancer increases mortality in people with T2DM in Lithuania is unknown.

Finally, it has been shown that the association between diabetes and mortality is different across countries and continents [11,12,13]. To our knowledge, no recent, reliable long-term data on mortality from specific causes of patients with T2DM are available in Lithuania or the Baltic region. 

The aim of this study was to analyze overall and cause-specific mortality risk in people with T2DM in Lithuania between 2010 and 2017 by comparing it with the general population of Lithuania.

## 2. Materials and Methods 

Cause-specific mortality risk among patients with diabetes in Lithuania was assessed using a retrospective cohort study design. Information on the diagnosis of T2DM and glucose-lowering medication was obtained from the National Health Insurance Fund (NHIF) database. Underlying causes of death were obtained from death certificates (Causes of Death Register). 

The NHIF database, created in 1999, contains demographic data and entries on all provided healthcare services, whether ambulatory or hospital-based, and prescriptions of reimbursed pharmacological agents. As the diagnoses are registered manually into the database by healthcare providers, to increase the specificity of diabetes cases, only patients who had more than 6 prescriptions for reimbursed glucose-lowering medications were included into the study. Furthermore, we analyzed only T2DM cases (International Statistical Classification of Diseases and Related Health Problems (ICD)-10 code E11), diagnosed at the age of 40 or older. 

In total, a cohort of 89,554 prevalent T2DM patients, who were alive on 01 January 2010, were identified from the NHIF database. Patients with the same recorded date of diagnosis and date of death (12 cases) and patients with missing information on cause of death (30 cases) were excluded from the cohort. Finally, 89,512 patients (32,611 men and 56,901 women) with T2DM were included in the analysis, contributing to the observation period of 592,321.4 person-years (212,050.1 and 380,271.3 for males and females, respectively).

Available data for this analysis included: Sex, age, date of diabetes diagnosis (first registration of diagnosis in the NHIF database) and date of death, underlying cause of death, and prescribed glucose-lowering medications. Cohort members were classified into three groups according to treatment: “Oral” medication users, “insulin and oral” medication users, and “insulin” users. According to the Lithuanian diabetes management guidelines, metformin is the initial oral glucose-lowering drug for T2DM. If metformin is contraindicated, causes side effects, or metformin monotherapy fails to achieve the glycemic goal (HbA1c less than 7%), the treatment is intensified by adding a second line medication, which is generally a sulfonylurea. As a third step, combination therapy with more than two classes of glucose-lowering drugs can be used (adding thiazolidinediones, glucagon-like peptide-1 analogs, or dipeptidyl peptidase-4 inhibitors). Insulin treatment can be initiated after failed combination therapy or at any time depending on the clinical situation. Metformin treatment is usually continued together with insulin [14].

To assess cause-specific mortality risk in the study cohort, common causes of death were classified into 10 broad categories and subcategories according to the ICD-10. The time since the diagnosis of diabetes was also stratified into three groups (1–5, 6–10, and >10 years). Patients with a first diabetes diagnosis between 1999 and 2000 included prevalent cases, therefore, they were excluded from the part of analysis by duration of follow-up (18,699 cases).

Follow-up started on 01 January 2010 and ended on 31 December 2017, the day of emigration, or the day of death, whichever came first. By 31 December 2017, 30,200 diabetic patients (11,829 men and 18,371 women) had died. Sex, age, and calendar period-standardized mortality ratios (SMRs) were calculated by dividing the observed number of deaths among patients with T2DM by the expected number of deaths, calculated using national rates. Assuming that data followed a Poisson distribution, 95% confidence intervals (CIs) for SMRs were calculated. The chi-square (χ^2^) test for trend was performed in order to evaluate changes in mortality risk of diabetic patients over age groups and time since diabetes diagnosis.

All statistical analyses were carried out using STATA 11 statistical software (StataCorp. 2009. Stata Statistical Software: Release 11.0. College Station, TX, USA). The study was conducted in accordance with the Declaration of Helsinki, and the protocol was approved by the Vilnius Regional Biomedical Research Ethics Committee (No. 158200-17-913-423).

## 3. Results

Baseline characteristics of the study cohort are presented in Table 1. 

An increased overall mortality risk was found for both sexes combined (SMR = 1.35, 95% CI 1.34–1.37). The SMRs were significantly increased in both male and female diabetic patients, with SMRs of 1.24 (95% CI 1.22–1.27) and 1.43 (95% CI 1.41–1.45), respectively (Table 2). The greatest mortality risk was for those in whom diabetes was diagnosed at a younger age (SMR = 1.68, 95% CI 1.60–1.76) and particularly high among those who had died before the age of 50 years (SMR = 22.04, 95% CI 18.82–25.81). The SMRs decreased with increasing age (test for trend *p* < 0.001) in both conditions, but remained significantly increased among those with T2DM who were aged 70 and over (SMR = 1.22). Excessive all-cause mortality risk was higher in females than in males, especially in the younger age group.

The risk for mortality increased with increasing time since diabetes diagnosis, with SMRs of 1.09, 1.23, and 1.36 in periods 1–5, 6–10, and >10 years after diagnosis, respectively (test for trend *p* < 0.001).

With regard to T2DM treatment groups (oral glucose-lowering drugs, insulin and oral, only insulin), significantly higher mortality risk than expected from the general population was found in all groups. The SMR varied depending on the disease treatment modality from 1.23 (95% CI 1.22–1.25) among those treated only with oral antidiabetic drugs to 2.43 (95% CI 2.32–2.55) among those treated with insulin only.

The observed and expected numbers and the SMRs for each specific cause of death by sex are shown in Table 3. The main causes of death among patients with T2DM were diseases of the circulatory system (65.2%), malignant neoplasms (15.3%), endocrine, nutritional and metabolic diseases (6.2% (mainly due to T2DM- 5.2%)) and diseases of the digestive system (4.3%). As compared to the general population, except for T2DM, the highest statistically significantly increased mortality risk was found for deaths from infection-related causes (SMR = 1.44), particularly septicemia (SMR = 1.78) and diseases of the circulatory system (SMR = 1.42), especially ischemic heart (SMR = 1.46) and cerebrovascular diseases (SMR = 1.38). However, the mortality risk ascribed to malignant neoplasms was slightly elevated only for female diabetic patients (SMR = 1.13), but not for males (SMR = 0.93), whereas mortality from all external causes and alcohol-related diseases was even lower in the diabetic cohort than in the general population—results for men were statistically significant.

## 4. Discussion

This nationwide study involved more than 89,000 people with T2DM, followed-up for over 592 thousand person-years, and over 30 thousand deaths were analyzed during the observational period. The large study cohort allowed us to investigate a wide range of mortality outcomes and compare it to the national population.

The study showed that people with T2DM had a 35% excess risk of mortality from all causes, with an even higher risk for women compared to men, especially in the youngest age group. Excess mortality in T2DM was substantially higher in people who were diagnosed with T2DM at a younger age, in those who died at a younger age, those who had a longer diabetes duration, or those who required treatment with insulin.

Furthermore, our study showed that both men and women with diabetes had not only an increased risk of death from diabetes itself and from CVD, but substantially higher mortality risk from infectious, digestive, and genitourinary diseases. The mortality risk due to cancer, however, was significantly elevated only for women with diabetes, probably because of increased corpus uteri and breast cancer risk that we have found in Lithuanian diabetic women [10]. Even more, increased risk for mortality from respiratory diseases was also seen in diabetic women.

Similar studies from across the world, both earlier [7,15,16,17] and recent [18,19,20,21], even with advances in diabetes treatment over the years, have also consistently shown that T2DM increases all-cause mortality; however, the association with nonvascular causes of death slightly differed among various populations. Interestingly, in our population, mortality from all external causes and alcohol-related diseases, as well as diseases from the nervous system, were lower in the diabetic cohort than in the general population. Data about these less-common causes of death in other studies are lacking and variable.

An analysis from the Verona Diabetes Study group of over 7 thousand T2DM patients in Italy, followed-up in 1987–1991, showed that diabetic patients had a 42% higher risk of mortality from all causes (compared to the general population). CVD diseases, cirrhosis, and diabetes contributed to increased mortality. As in our study, mortality risk from cancer was similar in the diabetic cohort and in the general population. Insulin treatment was strongly associated with mortality from all causes [7]. 

Moss et al. studied mortality risk for specific causes in both young onset and older onset (presumably T2DM) diabetes patient groups in Wisconsin, USA, between 1980 and 1988. The authors found similar results for mortality risk from CVD (SMR 2.3; 95% CI 2.1–2.5), diabetes (SMR 16.8; 95% CI 14-19.9), and malignancies (SMR 0.9, 95% CI 0.8–1.2). However, there was no risk association for mortality from external causes [15]. A Finnish study of death causes between 1981 and 1985 for people with drug-treated diabetes showed comparable results, with the youngest diabetes patients having greatest risk for increased mortality and diabetic women having higher excess risk than men [16].

A collaborative multinational diabetes mortality study from the UK, USA, and Northern Europe included data about base glucose levels and several risk factors from 97 prospective studies. In addition to vascular disease, diabetes was associated with premature death from several cancers, infectious diseases, external causes, intentional self-harm, and degenerative disorders, independent of major risk factors. Fasting glucose levels exceeding 5.6 mmol/L, but not normal glucose levels (3.9 to 5.6 mmol/L), were associated with higher risk for death [17].

Furthermore, a recently published study on data from a nationwide complex survey, NHANES, collecting health and nutrition data from the noninstitutional civilian United States population in 1999–2010 included 15,513 participants of which 2396 were diagnosed with diabetes (mainly type 2). The findings showed that diabetes at the baseline was associated with increased mortality risk due to CVD, chronic lower respiratory diseases, influenza and pneumonia, and kidney disease, but not with cancer or Alzheimer’s disease [18]. Another recent study from the USA’s national health interview data on mortality trends found that from 1988–1994 to 2010–2015, all-cause death rates declined by 20% every 10 years among US adults with diabetes, most in men and adults aged 65–74 years of age, but there was no decline in death rates among adults aged 20–44 years. The proportion of total deaths among adults with diabetes from vascular causes declined from 47.8 to 34.1%; however, this decline was offset by increases in the proportion of deaths from non-vascular, non-cancer causes, from 33.5% to 46.5%, suggesting upcoming implications for clinical management of diabetes patients [20].

A 24 year follow-up prospective study during 1991–2014 from the south of Sweden showed mortality risk to be increased by 47% in T2DM patients, with excess mortality mainly attributed to endocrine and cardiovascular cause of death with crude subdistributional hazard ratios of 5.06 (*p* < 0.001) and 1.22 (*p* < 0.001) [19].

With reference to CVD, it seems important to highlight the negative impact on diabetes survival attributable not only to ischemic heart and cerebral diseases, but also peripheral artery disease, often in combination with diabetic polyneuropathy, leading to lower-extremity amputations. It has been calculated that amputation is over 10 times as likely in those with diabetes as in those without diabetes [22]. Lower-extremity amputations are an important risk factor for mortality among diabetic patients, both for their complications and as a predictive factor, with 5 year mortality rates among patients with any amputation ranging from 53% to 100% [23].

While most of these epidemiological studies come from the western part of the globe, the trends of increased diabetes mortality do not seem to differ in the east, with the risk seeming to be even higher. A pooled analysis of 22 studies of the Asia Cohort found that people with diabetes had a 1.89 fold risk of all-cause death compared to patients without, with the highest relative risk of death due to diabetes (HR, 22.8; 95% CI, 18.5–28.1), followed by renal disease (HR, 3.08; 95% CI, 2.50–3.78), coronary heart disease (HR, 2.57; 95% CI, 2.19–3.02), and ischemic stroke (HR, 2.15; 95% CI, 1.85–2.51), and several types of cancer. Consistent with our findings, the adverse diabetes-mortality associations were more evident among women and younger adults [24].

As in our study, a meta-analysis that looked into sex differences in the association between diabetes and risk of CVD, cancer, and mortality of 49 prospective studies found that women had a 13% greater risk of all-cause mortality associated with diabetes, and there was a 30% significantly greater excess risk of CVD mortality in women with diabetes compared to men [25]. Interestingly, a study from Taiwan found that among patients with coronary artery disease, the impact of T2DM on mortality was consistently higher in women than in men, but the differences across sexes were not statistically significant after 1996, that is, after the wide application of coronary stents for CVD treatment [26].

With regard to diabetes treatment, we found that patients treated with insulin had a greater mortality risk than those treated with oral glucose-lowering medications or combination therapy. The association has been reported in other studies [7]. This may be related more to the severity of the disease than the treatment itself, as insulin is usually started when the disease cannot be managed by diet and oral medications alone or if there are contraindications for oral therapy, such as kidney or liver failure.

With regard to T2DM and risk for death from external causes, some studies found comparable risk [7,15,16] while others showed increased risk [17,27]; however, many studies have not reported deaths attributable to these factors. The inconclusive results warrant further investigation. It is most likely that these causes of death may be highly affected by social, economic, and cultural differences of the study populations.

Our study has strengths and limitations. The strengths of this study are the evaluation based on real-life data from well-established registries [28]. This study covers the whole population of Lithuania with a large sample size and long follow-up time, and the diagnosis of T2DM is well-defined. We even included diabetes treatment into our analysis. By linking data from several registries, we could identify all patients with pharmacologically treated T2DM and cover those that could have been missed by death registry analysis alone, for whom diabetes might not be listed as an underlying cause of death in the death certificate.

The limitations of our study are those that are common in cohort studies, such as shortage of clinical data such as body mass index and smoking status, or bias encountered with SMR calculation, where the true relative risk can be underestimated for relatively common diseases [29]. Furthermore, even though we have taken several measures to increase the specificity of identifying T2DM cases, there still remains a possibility that some patients, especially in the youngest age group, could have had type 1 or other types of diabetes, misclassified as T2DM. In addition, cause of death classification is generally a clinical decision of the doctor who fills in the death certificate. This could lead to bias in cause of death classification as deaths from CVD and kidney failure may be underestimated if part of these diagnoses ended up in the causes of death attributable to diabetes. Finally, some variables that are possibly associated with mortality (obesity, diabetes management, diet) may be different among countries; therefore, the results cannot be generalizable; however, they are a piece of the puzzle in the global picture.

## 5. Conclusions

In conclusion, people with T2DM in Lithuania had a 35% excess risk of mortality from all causes. Excess mortality was substantially higher in people who were diagnosed with T2DM at a younger age, died at a younger age, who had a longer diabetes duration, and those who required treatment with insulin. Women had a higher risk than men in all groups according to age, time after diagnosis, and therapy. Furthermore, both men and women with T2DM had increased risk of death from diabetes itself, cardiovascular, infectious, digestive, and genitourinary diseases. Women had an elevated mortality risk due to cancer and respiratory diseases as well. Therefore, excess mortality highlights the need for more effective measures to improve the outcomes of people with T2DM.

## Figures and Tables

**Table 1:** Characteristics of the study population.

	Men	Women	Overall
Patients with T2DM ^1^ (%)	32,611 (36.43)	56,901 (63.57)	89,512 (100)
Mean age at diabetes diagnosis (SD ^2^), yr ^3^	58.7 (9.99)	62.71 (10.03)	61.31 (10.19)
Mean age at death (SD), yr	73.36 (0.75)	79.03 (0.60)	76,81 (0.67)
Mean follow-up time (SD), yr	11.06 (3.69)	12.34 (3.65)	12.07 (3.68)
Mean diabetes exposure time (SD) *, yr	10.77 (3.32)	11.32 (3.26)	11.11 (3.29)
Number of deaths (%)	11,829 (39.17)	18,371 (60.83)	30,200 (100)

^1^ T2DM, type 2 diabetes mellitus, ^2^ SD, standard deviation; ^3^ yr, years; * prevalent diabetes cases at beginning of follow-up excluded from analysis.

**Table 2:** Standardized mortality ratios for all causes of death for men, women, and the overall cohort according to sex, age at diagnosis, age at death, time after diagnosis, and therapy.

	Men					Women					Overall				
	Obs ^1^	Exp ^2^	SMR ^3^	95% CI ^4^		Obs	Exp	SMR	95% CI		Obs	Exp	SMR	95% CI	
Overall	11,829	9512.57	1.24	1.22	1.27	18,371	12,849.93	1.43	1.41	1.45	30,200	22,362.50	1.35	1.34	1.37
Age at diagnosis															
40–49	1216	823.09	1.48	1.40	1.56	643	284.53	2.26	2.09	2.44	1859	1107.62	1.68	1.60	1.76
50–59	3026	2416.27	1.25	1.21	1.30	2643	1456.06	1.82	1.75	1.89	5669	3872.33	1.46	1.43	1.50
60–69	4267	3452.83	1.24	1.20	1.27	6339	4070.97	1.56	1.52	1.60	10,606	7523.81	1.41	1.38	1.44
≥70	3320	2820.37	1.18	1.14	1.22	8746	7038.38	1.24	1.22	1.27	12,066	9858.75	1.22	1.20	1.25
	*p* < 0.001					*p* < 0.001					*p* < 0.001				
Age at death															
40–49	116	6.12	18.97	15.81	22.75	38	0.87	43.64	31.76	59.98	154	6.99	22.04	18.82	25.81
50–59	1112	431.17	2.58	2.43	2.74	554	116.75	4.75	4.37	5.16	1666	547.93	3.04	2.90	3.19
60–69	2858	1873.01	1.53	1.47	1.58	2197	823.51	2.67	2.56	2.78	5055	2696.52	1.87	1.82	1.93
≥70	7743	7202.27	1.08	1.05	1.10	15,582	11,908.79	1.31	1.29	1.33	23,325	19,111.07	1.22	1.20	1.24
	*p* < 0.001					*p* < 0.001					*p* < 0.001				
Time after diagnosis *															
1–5	1506	1445.09	1.04	0.99	1.10	1748	1530.28	1.14	1.09	1.20	3254	2975.37	1.09	1.06	1.13
6–10	4321	3734.52	1.16	1.12	1.19	5945	4611.66	1.29	1.26	1.32	10,266	8346.18	1.23	1.21	1.25
>10	3025	2361.79	1.28	1.24	1.33	4886	3455.61	1.41	1.37	1.45	7911	5817.40	1.36	1.33	1.39
	*p* < 0.001					*p* < 0.001					*p* < 0.001				
Glucose-lowering drugs															
Oral	9329	8252.28	1.13	1.11	1.15	14,764	11,305.37	1.31	1.29	1.33	24,093	19,557.64	1.23	1.22	1.25
Insulin and oral	1650	872.50	1.89	1.80	1.98	2624	1178.46	2.23	2.14	2.31	4274	2050.96	2.08	2.02	2.15
Insulin	850	387.79	2.19	2.05	2.34	983	366.10	2.69	2.52	2.86	1833	753.89	2.43	2.32	2.55
			

^1^ Obs, observed; ^2^ Exp, expected; ^3^ SMR, standardized mortality ratio; ^4^ CI, confidence interval; * prevalent diabetes cases at beginning of follow-up excluded from analysis.

**Table 3:** Standardized mortality ratios for causes of death for men, women, and the overall cohort.

	Men					Women					Overall				
Diagnosis (ICD ^1^-10 code)	Obs ^2^	Exp ^3^	SM ^4^	95% CI ^5^		Obs	Exp	SMR	95% CI		Obs	Exp	SMR	95% CI	
Overall	11,829	9512.6	1.24	1.22	1.27	18371	12,849.9	1.43	1.41	1.45	30,200	22,362.5	1.35	1.34	1.37
Certain infectious and parasitic diseases (A00-B99)	172	143.1	1.20	1.04	1.40	248	148.8	1.67	1.47	1.89	420	291.9	1.44	1.31	1.58
Septicaemia (A40-A41)	127	73.5	1.73	1.45	2.06	196	108.4	1.81	1.57	2.08	323	181.9	1.78	1.59	1.98
Malignant neoplasms (C00-C97)	2169	2320.2	0.93	0.90	0.98	2437	2151.4	1.13	1.09	1.18	4606	4471.6	1.03	1.00	1.06
Endocrine, nutritional and metabolic diseases (E00-E88)	767	66.1	11.60	10.81	12.46	1114	122.6	9.08	8.57	9.63	1881	188.7	9.97	9.53	10.43
Non-insulin-dependent diabetes mellitus (E11)	618	50.5	12.25	11.32	13.25	953	98.3	9.70	9.10	10.33	1571	148.7	10.56	10.05	11.10
Mental and behavioural disorders (F01-F99)	22	20.5	1.07	0.71	1.63	52	41.2	1.26	0.96	1.66	74	61.7	1.20	0.95	1.51
Diseases of the nervous system (G00-G98)	79	115.7	0.68	0.55	0.85	149	174.3	0.85	0.73	1.00	228	290.0	0.79	0.69	0.90
Diseases of the circulatory system (I00-I99)	7086	5070.4	1.40	1.37	1.43	12,614	8770.5	1.44	1.41	1.46	19,700	13,840.9	1.42	1.40	1.44
Ischaemic heart diseases (I20-I25)	4926	3400.1	1.45	1.41	1.49	8277	5665.7	1.46	1.43	1.49	13,203	9065.8	1.46	1.43	1.48
Heart failure (I50)	35	38.1	0.92	0.66	1.28	88	72.1	1.22	0.99	1.50	123	110.2	1.12	0.94	1.33
Cerebrovascular diseases (I60-I69)	1506	1107.3	1.36	1.29	1.43	3323	2402.3	1.38	1.34	1.43	4829	3509.6	1.38	1.34	1.42
Diseases of arteries, arterioles and capillaries (I70-I79)	91	121.1	0.75	0.61	0.92	108	106.8	1.01	0.84	1.22	199	227.9	0.87	0.76	1.00
Diseases of the respiratory system (J00-J98)	355	438.7	0.81	0.73	0.90	315	256.8	1.23	1.10	1.37	670	695.5	0.96	0.89	1.04
Pneumonia (J12-J18)	112	129.9	0.86	0.72	1.04	136	105.7	1.29	1.09	1.52	248	235.6	1.05	0.93	1.19
Diseases of the digestive system (K00-K92)	540	436.8	1.24	1.14	1.34	753	518.2	1.45	1.35	1.56	1293	955.0	1.35	1.28	1.43
Diseases of liver (K70-K76)	220	185.1	1.19	1.04	1.36	169	125.1	1.35	1.16	1.57	389	310.2	1.25	1.14	1.39
Diseases of pancreas (K85-K86)	49	41.7	1.18	0.89	1.56	40	43.7	0.92	0.67	1.25	89	85.4	1.04	0.85	1.28
Diseases of the genitourinary system (N00-N98)	97	78.5	1.24	1.01	1.51	145	120.0	1.21	1.03	1.42	242	198.5	1.22	1.07	1.38
Disorders of kidney and ureter (N00-N28)	69	60.4	1.14	0.90	1.45	119	107.2	1.11	0.93	1.33	188	167.6	1.12	0.97	1.29
External causes of mortality (V01-Y98)	385	653.1	0.59	0.53	0.65	300	343.7	0.87	0.78	0.98	685	996.8	0.69	0.64	0.74
Accidents (V01-X59)	228	392.4	0.58	0.51	0.66	211	234.5	0.90	0.79	1.03	439	627.0	0.70	0.64	0.77
Suicides (X60-X84)	104	167.0	0.62	0.51	0.75	55	59.8	0.92	0.71	1.20	159	226.8	0.70	0.60	0.82
Event of undetermined intent (Y10-Y34)	32	60.6	0.53	0.37	0.75	26	30.7	0.85	0.58	1.25	58	91.3	0.64	0.49	0.82
Alcohol-related diseases*	94	157.0	0.60	0.49	0.73	52	65.5	0,79	0.61	1.04	146	222.4	0.66	0.56	0.77

^1^ ICD, International Classification of Diseases, ^2^ Obs, observed; ^3^ Exp, expected; ^4^ SMR, standardized mortality ratio; ^5^ CI, confidence interval, * F10, G312, G621, G721, I426, K292, K70, K852, K860, X45, X65, Y15.

## Abbreviations

T2DM	Type 2 diabetes mellitus
SMR	standardized mortality ratio
NHIF	National Health Insurance Fund
CI	confidence interval
CVD	cardiovascular disease
